# Trends in Multiple Health Complaints in Polish Adolescents in Light of Data from 30 European Countries and Canada (2002–2018)

**DOI:** 10.34763/jmotherandchild.20212501.d-21-00011

**Published:** 2021-10-11

**Authors:** Joanna Mazur, Helena Jeriček Klanšček, Lilly Augustine, Katarzyna Porwit, Erik Sigmund, Kastytis Šmigelskas

**Affiliations:** 1 Department of Humanization in Medicine and Sexology, University of Zielona Gora, Zielona Gora, Poland; 2 Department of Child and Adolescent Health, Institute of Mother and Child, Warsaw Poland; 3 Center for Analysis and Development of Health, National Institute of Public Health, Ljubljana Slovenia; 4 CHILD Research Group, School of Education and Communication, Jönköping University, Jönköping, Sweden; 5 Centre of Migration Research, University of Warsaw, Warsaw Poland; 6 Faculty of Physical Culture, Palacký University Olomouc, Olomouc, Czechia; 7 Health Research Institute, Faculty of Public Health, Medical Academy, Lithuanian University of Health Sciences, Kaunas, Lithuania; 8 Department of Health Psychology, Faculty of Public Health, Medical Academy, Lithuanian University of Health Sciences, Kaunas, Lithuania

**Keywords:** subjective complaints, mental health, trends, international data

## Abstract

**Background:**

Adolescence is a sensitive period accompanied by rapid developmental changes that can result in health complaints. The aim of the study was to describe the trend of subjective health complaints (HBSC-SCL) of Polish adolescents compared to their peers from 30 other countries and to rank all countries based on a proposed standardised measure.

**Material and Methods:**

Data from the Health Behaviour in School-Aged Children (HBSC) study collected from 2002 to 2018 were used. The overall number of respondents from 30 countries in the combined sample from five quadrennial rounds was 773,356, including 49.2% boys and 50.8% girls. The HBSC-SCL is a non-clinical measure consisting of eight health complaints, usually analysed in two dimensions of psychological and somatic symptoms. Linear regression analysis was applied to assess the significance of trends of the total index and two subindices in the combined sample and individual countries.

**Results:**

A significant increasing trend for the eight-item index appeared in Poland only in 13- and 15-year-olds, while only among 15-year-olds in the combined sample from 30 countries. Standardised country rank varied between -1.85 and 2.48 (worst). The countries that achieved extreme negative values (>=1) are France, Hungary, Italy, and Sweden, and the rank for Italy is considerably higher than for other countries. In Poland, the standardised rank for psychological symptoms exceeded the threshold of +1 in 2018.

**Conclusions:**

The HBSC-SCL index could be useful for monitoring change in adolescent mental health. The proposed method of ranking may allow a broader view of the differences and similarities between countries and help to identify those performing unfavourably against cross-country patterns.

## Introduction

Research has found that approximately one-third of youth report multiple health complaints at least once a week, which are, in turn, highly associated with poor mental health that commonly lasts into adulthood [1, 2, 3]. This issue seems to be especially prominent in girls, as internalising issues have increased over time in adolescent girls, but this relation is less clear in boys [4]. In addition, the trends regarding mental health seem to vary between countries, and national trends do not need to follow a general pattern [5].

Different factors seem to influence the development and/ or continuation of the symptoms, some being biological while others depend more on contextual factors and country characteristics. Previous literature has reported notable gender (i.e., girls reporting symptoms more often) [4, 5, 6, 7, 8] and age differences (i.e., older adolescents reporting increases in symptoms occurrence) [5, 6, 7]. Recurrent symptoms have been demonstrated to associate with health behaviours (e.g., earlier alcohol onset) [9, 10, 11], suicidal behaviour and ideation [12], and many other health and social problems.

The prevalence of psychosomatic symptoms differs substantially between countries, with some important factors being cultural and social norms, national policies, monitoring youth mental health, number of structural facilities and resources, social (in)equality, investment in family benefits and education, country GNI, etc. [6, 13, 14, 15, 16]. Factors associated with health complaints are more related to the proximal environment (family, peers, schools, regions) than to distal macro-level factors, which points toward intensifying targeted interventions and also targeting specific risk groups [6]; nevertheless, some macro-level impact suggests that some changes (e.g., country`s increase in wealth) might actually improve health in a larger population [7].

Health complaints are important for the assessment of adolescent health, both physical and mental. The adolescent complaints list was elaborated within the Health Behaviour in School-Aged Children (HBSC) scale for use in three age groups (11, 13, and 15 years) and includes eight symptoms: four psychological (feeling low, irritability or bad mood, feeling nervous, and dizziness) and four physical (headache, abdominal pain, backache, and sleeping difficulties). Validation studies conducted in recent years show sufficient internal consistency and also comparability of findings using online or paper mode [17]. This scale has been validated not only in Europe but also in other countries such as Canada [18] and China [19].

The prevalence of subjective complaints among young people aged 11 to 15 years may be compared on an international scale by analysing successive international HBSC reports [20]. The ranking of countries is presented in graphic form for each of the three age groups, split up into boys and girls, but the country’s place in the ranking is determined by an overall indicator for the given age group. The changing age structure in individual countries and survey years creates a methodological problem, which confirms the rationale for examining each age group separately instead of using one indicator for the combined age group of 11 to 15 years. Data relating to Poland confirm that its place in the ranking is moving in an unfavourable direction in the 11 to 15 age group and that it is also worse for girls than for boys. Moreover, it is difficult to draw unequivocal conclusions about the changing place of a country in the ranking in time because the number of members of the HBSC network is constantly increasing. In light of the available knowledge, no attempt has been made to develop a standardised international ranking that would take into account the changing position of a given country in successive age groups as well as the level of the total index and partial indices at the same time. Standardised rank could help to assess the actual distance between countries and identify countries with the worst and the best results. This methodological approach also sheds new light on trend analyses.

The study aims to draw attention to non-specific subjective complaints as an important health issue among school children and youth. A trend analysis was carried out for 2002 to 2018, based on data from 30 countries, presenting Polish data on the backdrop of international statistics, with an interpretation of general tendencies and considering partial complaint indices. In the methodological section, the advantages of using standardised rank as a measure of distance between countries will be discussed.

## Material and methods

### Sample and weighting

The results refer to school children ages 11, 13, and 15 years surveyed in five successive rounds of HBSC studies [20]. Thirty countries have qualified for the analyses, which possessed available data for all analysed symptoms during the school years 2001–2002, 2005–2006, 2009–2010, 2013– 2014, and 2017–2018. The international data file developed and provided to member states by the HBSC International Data Bank in Bergen was used. The overall number of respondents from the 30 countries in the combined sample from five rounds was 773,356, including 49.2% boys and 50.8% girls. The average age of respondents was 13.55 (SD=1.64) years, and the share of individual age groups (school grades) was as follows: 11 years, 32.9%; 13 years, 34.4%; and 15 years, 32.7%. Schools acted as the primary sampling unit. The school response rate among the survey cycles varied from 75 to 99%, and the adolescent participants’ response rate mostly exceeded 80%.

Data from all the countries were collected according to a unified procedure developed in protocols assigned to successive rounds of the study. The governing principle was to conduct an anonymous survey in schools during class hours. The countries applied individually for approval by the relevant ethical committee in accordance with applicable national regulations. In Poland, the HBSC study procedure and ethical considerations were described in successive international reports. Since 2006, a rule requires parents to give their consent for a child to participate in a study, and the Bio-ethical Committee of the Institute of Mother and Child in Warsaw must issue an opinion and approve the contents of the questionnaire as well as the research procedure and informed consent (opinion nr 17/2017 issued on March 17, 2017, for 2017–2018 survey).

The number of respondents in successive rounds of HBSC studies, their average age, and the size of national samples varied by country. The recommended size of the national sample in each round is 4,500 students (1,500 in each age group). However, some countries exceeded this number considerably in at least one round. More than 8,000 cases were examined in at least one round in France, Spain, Russia, Czechia, and Canada. Traditionally, the smallest sample is from Greenland, which has the status of a separate region, and being a small region, it tries to reach the entire population of students from this age group. Individual countries differed in terms of the average age of respondents due to the varied participation of individual age groups in successive rounds of the study and different rules for the admission of pupils to the school grades.

The Polish sample numbered from 4,262 in 2010 to 6,383 in 2002, and surveys were conducted in all 16 voivodships. Only in 2006 were all the age groups sampled from 12 voivodships, but 15-year-olds were also sampled in the remaining four. As a result, in 2006 the participation of 15-year-olds was relatively higher, as was the average age of the entire Polish sample. Similar discrepancies in the age structure of those polled may occasionally be observed in many other countries.

To simplify the analyses to one indicator per country and level out the above limitations, samples from various countries and study periods were subjected to a weighting procedure. A hypothetical sample size was assumed for each country and study round, which was equal to 5,136 students, consisting equally of 856 students in six groups distinguished by gender and age group. As such, the hypothetical general sample from five study rounds is 770,400 persons, which does not significantly diverge from the actual sample.

### Measures

The scale of subjective complaints (HBSC-SCL stands for “subjective complaints checklist”) has been applied in international HBSC studies from the beginning of the network’s research activity. It was modelled on Norwegian studies [21]. In its extended version, 15 or 11 symptoms may be analysed. From the theoretical point of view, complaints involving pain (headache, stomach ache, back pain) and dizziness have a physical (somatic) nature associated with the function of specific organs. Negative emotional states (depression, irritation or bad mood, nervousness, and sleep difficulties), on the other hand, create a uniform group of symptoms with a strong mental background. Further in the study, these two groups of symptoms will be referred to as somatic and psychological complaints.

When answering the question about the occurrence of each symptom, young people were asked to assess its intensity over the previous six months by giving one of five answers ranging from “nearly every day” to “rarely or never.” In the present study, we built a general index by adding the responses and obtaining a summary scale of 0 to 36 points. Partial indices of somatic and psychological complaints range from 0 to 16 points. Answers were re-coded in such a way that a higher score indicates a more frequent occurrence of health problems.

In the analysed sample of 700,000 teenagers, Cronbach’s alpha was 0.805 for the general index and 0.687 and 0.739 for the somatic and psychological complaints indices, respectively. For the entire sample, the homogeneity of the scale was confirmed using principal component analysis (PCA), the main factor accounting for 42.8% of overall variability. In case of partial indices of somatic and psychological complaints, the percentage of explained variability is even higher (51.9% and 56.8%, respectively).

### Statistical analysis

With the help of weighted data, average values (with SD) of the general and partial HBSC-SCL indices for the successive study rounds were provided. A linear trend of these average values was estimated, giving parameters representing the time variable (study rounds) and goodness-of-fit statistics (R-squared coefficient). The trend analysis covered only the combined sample from 30 countries and Poland. A standardised rank of each country on the backdrop of the average values in all countries was developed, taking as the basis data from six partial age/gender groups. Rank was estimated using the regression method with the help of PCA. Rank is a standardised z-score measure with a 0 average and standard deviation of 1. High values were obtained by countries where the intensity of subjective complaints was higher and lower values by countries where young people reported such complaints less frequently. This type of approach is considered an alternative taxonomical method, which enables the determination of the distance between countries based on various indicators. Separately, ranks for each country and for each of the five study periods were also defined (data not presented). In this case, the average is 0, and the standard deviation is 1 in a given year.

## Results

Table 1 presents the trend of average general HBSC-SCL indices in Poland and in the combined international sample, taking into account gender and age group. In the case of the general group, a stronger trend is observed in Poland than in the sample of 30 countries. A significant increasing trend is observed in Poland in both genders. In the international sample, a stronger growing trend was observed in the case of girls than boys, although the result is on the border of statistical significance (p=0.047). For boys, the average indices did not change significantly during the years 2002 to 2014, and the increase applied only to 2018. Conclusions concerning the three age groups are also different. An important increasing trend of the average HBSC-SCL value appeared in Poland only in the two older age groups and in the combined sample from the 30 countries only among 15-year-olds. The average HBSC-SCL mean index proved to be stable during the years 2002 to 2018 in the youngest age group in both Poland and the international sample.

**Table 1:** Mean level of a general HBSC-SCL index with trend analysis (weighted data).

Sample	Mean HBSC-SCL indices ± SD					Linear trend	
	2002	2006	2010	2014	2018	p	R-sq
**Total 30 countries Poland**	7.69±6.11 7.89±6.27	7.59±6.20 7.97±6.34	7.64±6.34 8.38±6.83	7.97±6.69 8.91±7.76	8.62±6.63 9.08±6.45	0.078 0.004	0.698 0.954
**Boys 30 countries Poland**	6.62±5.70 6.76±5.84	6.52±5.74 6.84±5.85	6.54±5.90 7.22±6.48	6.59±6.04 7.45±7.21	7.24±5.93 7.65±5.72	0.210 0.002	0.457 0.972
**Girls 30 countries Poland**	8.74±6.32 9.00±6.48	8.64±6.45 9.09±6.61	8.72±6.56 9.54±6.96	9.33±7.01 10.34±8.02	9.97±6.99 10.48±6.81	0.047 0.008	0.780 0.931
**11 yrs 30 countries Poland**	6.85±6.04 7.17±6.28	6.54±5.97 7.05±6.39	6.57±6.06 7.31±6.29	6.56±6.16 7.04±6.95	7.21±6.06 7.53±5.91	0.613 0.353	0.095 0.286
**13 yrs 30 countries Poland**	7.69±6.04 7.72±6.15	7.63±6.15 8.02±6.32	7.69±6.34 8.25±7.14	8.02±6.68 8.97±8.00	8.71±6.60 9.17±6.38	0.061 0.003	0.742 0.960
**15 yrs 30 countries Poland**	8.50±6.14 8.76±6.27	8.57±6.31 8.82±6.20	8.63±6.43 9.58±6.83	9.28±6.90 10.69±7.86	9.89±6.91 10.53±6.69	0.023 0.019	0.863 0.878

If we look separately at the trend of somatic and psychological complaints in the entire group without division into gender or age, an important trend appears in the international sample from 30 countries with regard to somatic complaints. In the same international sample, complaints with a stronger mental background remained at a stable level during the years 2002 to 2010 and only began to intensify from 2014 onward. Conversely, in Poland, the average index of somatic complaints increased during the years 2002 to 2014, only to decline in 2018. This is an opposite trend in comparison with the index of psychological complaints, which increased during the years 2014 to 2018 after a period of relative stability (Table 2).

**Table 2:** Mean level of partial HBSC-SCL indices with trend analysis (weighted data).

Complaints/ country	2002	2006	2010	2014	2018	p-trend	R-sq
**Somatic 30 countries Poland**	3.01±3.16 2.67±3.17	3.00±3.19 2.85±3.22	3.13±3.17 3.29±3.51	3.20±3.20 3.65±4.00	3.24±3.18 3.05±3.07	0.009 0.242	0.924 0.413
**Psychological 30 countries Poland**	4.67±3.79 5.20±4.02	4.56±3.82 5.09±3.99	4.50±3.85 5.07±4.14	4.75±4.07 5.23±4.55	5.30±4.13 5.98±4.28	0.166 0.181	0.526 0.500

In Table 3, average overall and partial indices in the 30 countries are compared, with an indication of the standardised rank of each country. The data are based on a combined sample from five rounds of HBSC studies. The average general HBSC-SCL index varied between 6.03 in Portugal and 10.43 in Italy. The average index of somatic complaints varied between 2.22 and 4.16, assuming extreme values in the same countries. In the case of an average index of complaints with a stronger mental background, the lowest value was recorded in Austria (3.64) and the highest in Italy (6.24). Standardised country rank varied between -1.85 and 2.48 for the general HBSC-SCL index. The countries that achieved extreme values (>=1) are France, Hungary, Italy, and Sweden, where the value of the rank for Italy is considerably higher than for the other countries. Austria, Greenland, Holland, Portugal, and Slovenia are in a privileged situation (rank <=-1). In the case of somatic complaints, it is also worth taking a look at Belgium (Francophone region) and Ukraine, which have a relatively less favourable position in the ranking. In terms of psychological complaints, the standardised country rank also exceeds the +1 level for Czechia and Greece. In Poland, the average rank was 0.54 for the general HBSC-SCL index and was much more favourable for somatic than for psychological complaints. In the first case, the figure was =-0.05, which is below the international average; in the second case, it was 0.89, which is close to the limit considered disturbingly high.

**Table 3:** Comparison of 30 countries according psychosomatic complaints based on combined data 2002–2018.

Country	Mean HBSC-SCL index based on weight data			Mean standardized country rank		

	Total	Somatic	Psycholo-gical	Total	Somatic	Psycholo-gical
**Austria**	6.34±6.02	2.68±3.13	3.64±3.57	-1.49	-0.93	-1.70
**Belgium (Flemish)**	6.91±5.94	2.81±3.12	4.07±3.60	-0.93	-0.64	-1.02
**Belgium (French)**	8.76±6.73	3.63±3.47	5.10±4.13	0.86	1.13	0.57
**Canada**	8.39±6.62	3.45±3.43	4.91±3.94	0.48	0.75	0.25
**Croatia**	7.39±6.18	2.78±3.08	4.58±3.93	-0.51	-0.74	-0.29
**Czechia**	8.62±5.98	3.08±2.95	5.52±3.80	0.74	-0.06	1.25
**Denmark**	7.17±5.64	2.74±2.92	4.40±3.54	-0.70	-0.80	-0.51
**Estonia**	8.15±6.67	3.22±3.30	4.91±4.18	0.26	0.26	0.25
**France**	9.15±6.28	3.73±3.23	5.38±3.95	1.22	1.33	1.00
**Germany**	7.13±5.77	3.19±3.13	3.92±3.38	-0.73	0.16	-1.27
**Greece**	8.32±6.43	2.71±3.14	5.58±4.24	0.41	-0.88	1.25
**Greenland**	6.70±6.66	2.62±3.37	3.99±4.13	-1.23	-1.09	-1.31
**Hungary**	8.98±6.58	3.75±3.43	5.20±3.96	1.08	1.39	0.73
**Ireland**	7.43±6.24	2.88±3.14	4.49±3.82	-0.45	-0.45	-0.42
**Italy**	10.43±6.46	4.16±3.47	6.24±4.03	2.48	2.24	2.35
**Latvia**	8.30±6.56	3.15±3.35	5.12±4.01	0.38	0.06	0.57
**Lithuania**	8.00±6.93	3.00±3. 51	4.98±4.25	0.08	-0.24	0.30
**Netherlands**	6.52±5.87	2.55±3.14	3.94±3.53	-1.33	-1.21	-1.22
**Norway**	7.52±5.96	2.94±3.09	4.55±3.61	-0.36	-0.38	-0.30
**Poland**	8.44±6.77	3.10±3.43	5.31±4.12	0.54	-0.05	0.89
**Portugal**	6.03±6.01	2.22±2.93	3.79±3.86	-1.85	-1.96	-1.55
**Russia**	7.46±6.90	3.20±3.57	4.24±4.10	-0.41	0.20	-0.81
**Slovenia**	6.51±5.97	2.26±2.85	4.21±3.83	-1.36	-1.81	-0.88
**Spain**	6.98±6.51	2.84±3.38	4.10±3.94	-0.90	-0.60	-1.04
**Sweden**	9.38±6.39	3.69±3.33	5.65±3.77	1.42	1.26	1.39
**Switzerland**	8.36±5.86	3.31±3.07	5.04±3.60	0.48	0.44	0.51
**Ukraine**	8.85±6.28	3.70±3.26	5.12±3.83	0.92	1.23	0.57
**England**	8.68±6.61	3.53±3.34	5.12±4.03	0.78	0.92	0.60
**Scotland**	7.76±6.45	3.05±3.20	4.69±3.98	-0.13	-0.13	-0.10
**Wales**	8.16±6.57	3.39±3.30	4.73±4.00	0.25	0.59	-0.04
**Total**	7.90±6.42	3.11±3.27	4.75±3.95	0.00	0.00	0.00

Based on 2002-2018 data, countries can be classified into five groups according to standardised rank and presented within the groups in order from very favourable to very unfavourable position in the ranking of countries:

- Very favourable level of HBSC-SCL (rank lower than -1): Portugal, Austria, Slovenia, Netherlands, Greenland
- A rather favourable level of HBSC-SCL (-1 to -0.5): Belgium (Flemish), Spain, Germany, Denmark, Croatia
- The average level of HBSC-SCL (-0.5 to +0.5): Ireland, Russia, Norway, Scotland, Lithuania, Wales, Estonia, Latvia, Greece, Switzerland, Canada
- A rather unfavourable level of HBSC-SCL (+0.5 to +1): Poland, Czechia, England, Belgium (French), Ukraine
- Unfavourable level of HBSC-SCL (rank above +1): Hungary, France, Sweden, Italy

If analogous ranks were calculated separately for each round of HBSC studies (Table 4), from the point of view of the overall HBSC-SCL index and the index of somatic complaints for Poland, the year 2014 was critical. Conversely, in 2018 Poland’s position clearly deteriorated in terms of complaints with a mental background (rank 1.02).

**Table 4:** Standardized rank for Poland based on year specific ranking.

Complaints	2002	2006	2010	2014	2018
**Total HBSC-SCL**	0.166	0.314	0.643	0.917	0.477
**Somatic**	-0.671	-0.286	0.302	0.993	-0.432
**Psychological**	0.745	0.716	0.846	0.754	1.022

**Figure 1:**
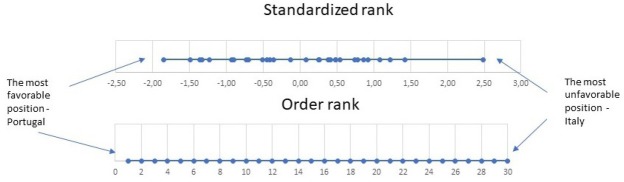
Comparison of ordered and standardized country rank.

Figure 1 presents a graphic presentation of the difference between a new standardised rank and the traditional one resulting from ordering all countries under study. The advantage of a standardised rank is the inclusion of the age and gender factor in its construction. Differences and similarities between countries are more visible, and it is easier to assess when a country scores very high compared to others.

## Discussion

The paper describes trends in psychosomatic complaints among adolescents in Poland compared to 30 countries, using data from the HBSC surveys. Such analyses were repeated periodically, taking into account successive new time points [4, 5, 7, 22, 23]. According to the recent HBSC publication, from 2002 to 2018 we could observe fairly stable upward or downward trends in health complaints in most countries, with a small overall linear increase over this period [5]. Our paper adopts a different analytical strategy (country inclusion criteria, weighted data, concentration on ranking, gender and age factor included in the rank) and shows how the data can be interpreted from the perspective of a selected country.

The added value of our paper is clustering the countries according to a standardised position. The groups according to standardised rank are extremely heterogeneous, and to the best of our knowledge, we could not observe any concrete connections among countries in the same group that would be explained by previous research. Countries in the same group differ in GDP, national policies regarding (mental) health of adolescents, investment strategies, gender equality, social and income (in)equality, etc. We could not pinpoint an exact source for the observed results, and further research on the topic is needed so that targeted changes can be made to improve the level of HBSC-SCL across the globe.

However, more attention should be paid to countries that are in a very unfavourable position in relation to others. Sweden can serve as an example. In Sweden, girls, especially at 15 years of age, and their mental health problems have been described as a paradox [24]. High levels of mental health problems in teenagers have been thoroughly investigated [25], and aspects of school and vocation are usually considered the reasons. School climate [26] and school pressure [5, 14, 27, 28] are usually considered the driving forces behind these mental health complaints, especially in girls. This increase in mental health symptoms was highlighted in the 1990s in the wake of a financial crisis in Sweden; however, even if the mental health did improve some during the early 2000s, it did not go back to the levels it had been at before, and when comparing it with, for example, the 1970s, we can see a reduction in mental health especially in girls [29]. In Sweden, there is also an increase over time in depression and anxiety diagnoses within the teenage group, especially for girls [30]. This increase is not seen within other age groups, indicating issues with this group.

Given the purpose of this paper, more attention should also be paid to Poland, which is highlighted in this study. The HBSC surveys are a notable source of information on trends in the prevalence of psychosomatic complaints and their determinants, as evidenced by previous national publications [31, 32]. Much attention was also paid to students with chronic conditions, treating subjective complaints as an additional and often ignored burden [33]. National analyses or those initiated by the Polish HBSC team aim to highlight protective factors against psychosomatic complaints, including behavioural factors and those related to support in the family and peer group [34, 35, 36]. Poland is an example of a country that comes alarmingly close to those in which psychosomatic complaints are more frequent. Although, as said above, there is no unambiguous geographical distribution of the participating countries according to multiple health complaints in summary from 2002 to 2018, the Visegrad 4 countries characterise the rather unfavourable level of HBSC-SCL (Poland, Czechia) and definitely the unfavourable level of HBSC-SCL (Hungary). Complete 2002-2018 data for Slovakia were not available, but the significant scale of the problem was confirmed by 2018 data. The benefits of presenting a change in ranking position in successive years are indisputable, as confirmed by the analysis carried out for Poland (Table 4). Other countries also changed their position significantly. For example, in France, for the entire 2002–2018 period, the standardised rank exceeded +1, placing this country in the risk group. In 2018, however, a favourable downward shift in the international ranking could be observed. The significantly higher prevalence of subjective complaints in Poland than in Portugal (the leading country in our ranking) was confirmed in a joint research and intervention project aimed at improving adolescent mental health [37].

Results obtained for Poland correlate with reports of a general crisis in child psychiatry. There is also talk of a breakdown in the system of psychological and psychiatric care for the developmental age population. At the core of the reform just implemented by the Ministry of Health is the creation of a reference system of level I, II, and III institutions. Level I institutions are to ofer psychological and psychotherapeutic assistance in the child’s environment. According to Professor Maciej Pilecki, level I institutions are the place where an experienced psychologist or psychotherapist will decide whether a young person needs specialist psychiatric consultation at all [38].

Finally, it is worth highlighting the strengths and weaknesses of the analyses presented. Strict adherence to the research protocol and standardised questionnaire and large sample size with high response rates in all the quadrennial survey cycles are the major strengths of the presented trend-related study. However, as a limitation, it is worth emphasising that the total scoring of complaints suggests that every symptom has the same value as the general score, though the symptoms may not be of the same weight for adolescent health or wellbeing. Differences in trends of somatic and psychological complaints were shown. Analogous findings could be made about individual symptoms. In addition, in the measurement of health complaints, there is no general agreement about cut-of points for clinically or practically significant diferentiation. Moreover, the potential efect of social desirability or the degree of diligence in filling out the questionnaire could influence these answers. However, anonymity, the possibility of omitting questions, and the impossibility of comparing answers with classmates/peers reduce the potential impact of social desirability.

Taking into account previous studies on methodological approaches in trend analyses based on the international HBSC data, our study can be classified as “the stratified approach,” as the significance of changes in each of the 30 countries was shown separately. When results in the combined sample from 30 countries were presented, advanced statistical methods (country*time point interaction, multilevel models) were ignored. However, the data weighting rule was applied, which, in light of Schnohr et al., is an important (but seldom used) step to increase the comparability of data collected at diferent times and in diferent countries [39]. An original methodological approach of a standardised rank was used, which referred to the graphs in the international HBSC reports but summarised age/gender differences in one measure [20]. To conclude, this approach seems to be easy to interpret and useful from a specific country perspective and can be recommended for further studies. Given the range of information about adolescent health available from HBSC surveys and similar projects, a similar analytical strategy could be applied for international comparisons of other indices, not only the HBSC-SCL index.

## Key points

- The HBSC-SCL index is an important indicator for monitoring change in adolescent mental health.
- The proposed method of ranking may allow a broader view of the differences and similarities between countries and help to identify those performing unfavourably against cross-country patterns.
- At present, mental health problems are increasing in Poland, and the level of the relevant subindex deviates unfavourably from international data.
